# MicroRNA expression profiles of serum from patients before and after chemotherapy

**DOI:** 10.1016/j.gdata.2015.08.018

**Published:** 2015-09-02

**Authors:** Yvonne Diener, Thomas Walenda, Edgar Jost, Tim H. Brümmendorf, Andreas Bosio, Wolfgang Wagner, Ute Bissels

**Affiliations:** a Miltenyi Biotec GmbH, Bergisch Gladbach, Germany; b Helmholtz Institute for Biomedical Engineering, RWTH Aachen University Medical School, Aachen, Germany; c Department for Hematology, Oncology, Hemostaseology and Stem Cell Transplantation, RWTH Aachen University Medical School, Aachen, Germany

**Keywords:** MicroRNA, Hematopoietic stem cells, Serum, Microarray

## Abstract

Recovery of the blood and immune system after chemotherapy requires proliferation of hematopoietic stem and progenitor cells (HPSCs). It has been shown that systemically released factors in serum after chemotherapy stimulate HSPC expansion in vitro. We wondered if microRNAs (miRNAs) circulating in serum could account for this effect. Therefore, we compared the miRNA expression profiles of serum from patients with hematologic malignancies before and after chemotherapy. In addition to a general decrease in miRNA expression after chemotherapy, we found 23 miRNAs to be significantly differentially expressed in serum before versus after chemotherapy. The miRNA microarray data are available at NCBI's Gene Expression Omnibus (GEO) Series accession number GSE57570. Here, we provide a detailed protocol of the miRNA microarray and data analysis.

Specifications

**Table:** 

Organism/cell line/tissue	*Homo sapiens* (serum)
Sex	Male and female
Sequencer or array type	Agilent Human miRNA Microarray Release 16.0, 8 × 60 K
Data format	Raw and processed
Experimental factors	Serum before vs. after chemotherapy
Experimental features	Profiling of circulating microRNAs in serum from patients with different hematologic malignancies before and after chemotherapy as well as healthy donors
Consent	Allowed for reuse citing original authors
Sample source location	Aachen, Germany

## 1 Direct link to deposited data

http://www.ncbi.nlm.nih.gov/geo/query/acc.cgi?acc=GSE57570

## 2 Experimental design, materials and methods

### 2.1 Experimental design

Chemotherapy and hematopoietic stem cell transplantation (HSCT) are standard therapies for the treatment of hematologic malignancies. The subsequent recovery of the blood and immune system requires activation and proliferation of hematopoietic stem and progenitor cells (HSPCs). Walenda and colleagues [1] demonstrated that systemically released factors in serum from patients after chemotherapy stimulate in vitro expansion of HSPCs. MicroRNAs (miRNAs) have been found to circulate in serum and their expression levels vary in physiological and pathological conditions [2], [3]. Furthermore, they are important regulators of cell fate and are able to influence hematopoietic stem cell proliferation and differentiation [4]. In this study [5], we have compared the miRNA expression profiles of serum from nine patients with lymphoma, acute myeloid leukemia (AML) or multiple myeloma (MM) before and after therapy as well as seven healthy donors as control. In summary, the presence of 1205 human and 144 human viral miRNAs was analyzed using the Agilent Human microRNA Microarray platform (Rel. 16).

### 2.2 Sample preparation

Serum samples from seven healthy donors and nine patients with different hematologic malignancies were collected at different time points in the course of therapy after informed written consent as described before [1]. Ten milliliters of peripheral blood was collected before chemotherapy and during leukopenia after chemotherapy and transferred into a 15 mL tube (Greiner). For preparation of serum, the blood was agitated horizontally at 37 °C for 1 h to allow coagulation and incubated upright at 4 °C for 4 h. After centrifugation for 15 min at 840 ×* g*, the serum supernatant was carefully removed, aliquoted and stored at − 80 °C until use. Further information on serum samples and patients was published by Walenda et al. [5]. For total RNA extraction, 500 μL of frozen serum was thawed for 30 min at room temperature and mixed with three volumes of TRIzol LS Reagent (Ambion). RNA was isolated using the miRNeasy mini kit (Qiagen) according to the manufacturer's instructions. The volume of the obtained RNA solution was reduced in a Speed Vac vacuum concentrator to a final volume of 1 μL.

### 2.3 RNA labeling and microRNA microarray hybridization

The Human microRNA Microarray Kit (Rel16.0, Agilent Technologies) was used for labeling and hybridization according to the manufacturer's protocol. In brief, 1 μL of total RNA was labeled with Cyanine 3 (Cy3), resuspended in hybridization buffer and hybridized to the array platform overnight (20 hours) at 55 °C in a rotating Agilent hybridization oven using Agilent's recommended hybridization chamber. Subsequently, the microarrays were washed with the Agilent Gene Expression Wash Buffer 1 for 5 min at room temperature. A second washing step was performed with Agilent Gene Expression Wash Buffer 2 warmed to 37 °C for 5 min. Fluorescence signals after hybridization were detected with a DNA microarray scanner G2505C (Agilent Technologies) using one color scan setting for 8 × 60 K array slides (Scan Area 61 × 21.6 mm, Scan resolution 3 μm, Dye channel is set to Green and Green PMT is set to 100%).

### 2.4 Microarray data analysis

**Fig. 1:**
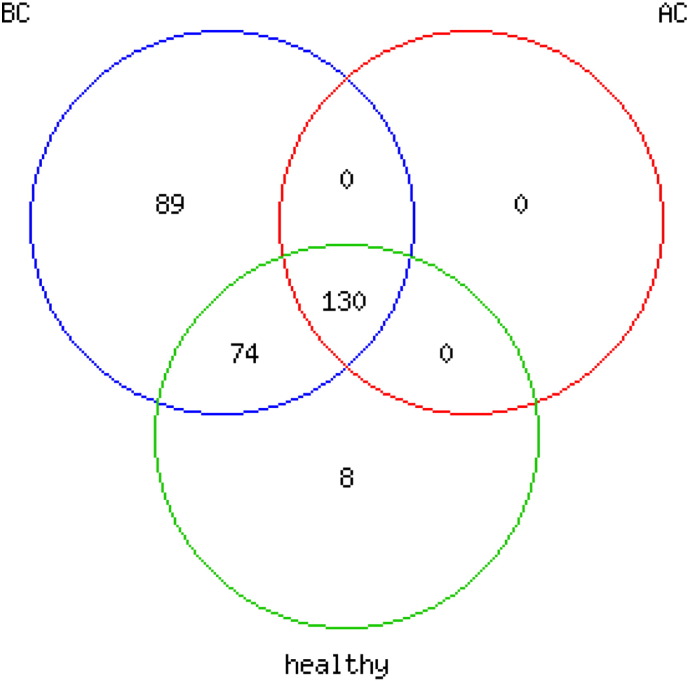
Higher number of detected microRNAs in patients' sera before chemotherapy. VENN diagram presenting numbers of detected miRNAs in serum from healthy donors (healthy), patients before (BC) and after chemotherapy (AC). Total gene intensities were filtered for miRNAs with median expression ≠ 0.1 over all donors. Exclusively in healthy donors detected miRNAs (8) were either detected with low signal intensities (< 10) or characterized as viral RNAs (HSV and KSHV).

**Fig. 2:**
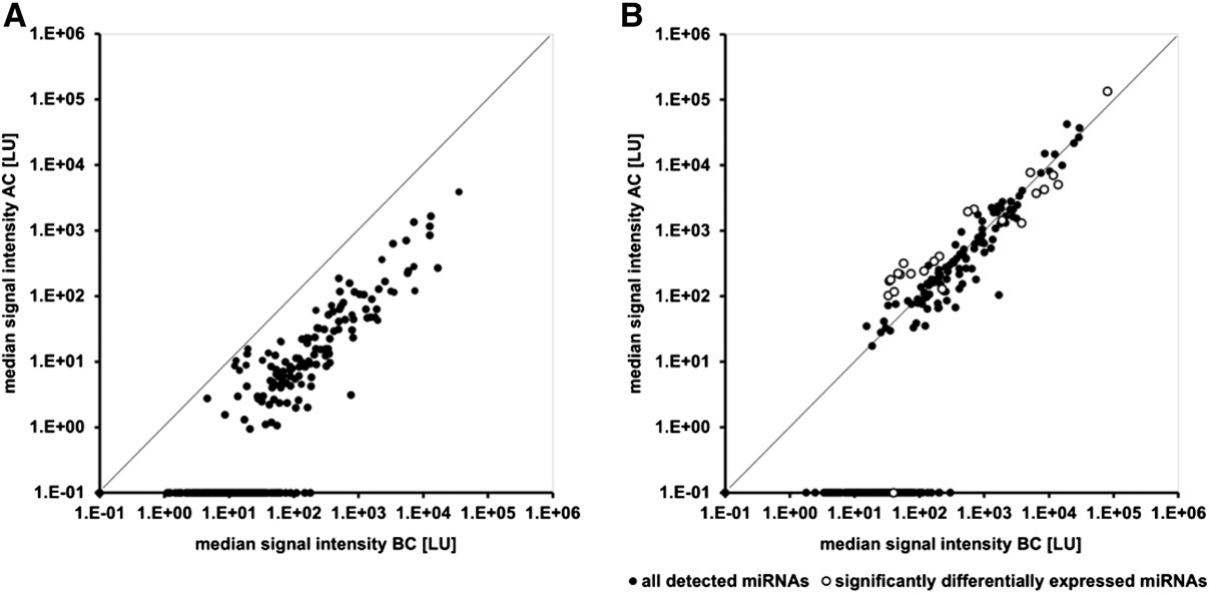
Median normalization of miRNA microarray data enabled identification of significantly differentially expressed miRNAs. Median signal intensities of miRNAs in serum after (AC) versus before chemotherapy (BC, n = 9), before (A) and after median normalization (B). White dots represent significantly differentially expressed microRNAs after normalization. Data points on the x-axis represent 163 miRNAs that are exclusively detected in BC serum.

In order to obtain background subtracted and outlier rejected signal intensities, the scanned microarray images were analyzed and processed with the Agilent feature extraction software (v10.7.3.1) using default parameters (protocol miRNA_107_Sep09 and Grid: 031181_D_F_20111226). The resulting raw Total Gene Signal intensities (SI, gTotalGeneSignal column) were exported to Microsoft Excel and filtered for detected miRNAs (SI ≠ 0.1). Analysis of the detected miRNAs revealed a lower number of miRNAs in serum taken after chemotherapy compared to serum before chemotherapy (Fig. 1) as well as decreased miRNA signal intensities after chemotherapy (Fig. 2A). This global decrease in miRNA expression levels significantly correlated with leucocyte numbers in the patients' blood, which might indicate that the observed alterations are caused by leukopenia. As we were interested in miRNAs significantly differentially expressed in serum before versus after chemotherapy independently from the disturbance in blood cell counts, the data were normalized to the array median as follows: (1) a filter was set for miRNAs which were detected with an SI ≠ 0.1 in all patient samples before and after chemotherapy (= 18 samples), giving 66 miRNAs in total (including Agilent positive controls: dmr, hur). (2) For each sample, the median over these 66 miRNAs was calculated. (3) The value of each single miRNA was divided through the second highest median value. The highest median value was not used as it presumably resulted from an outlier. For statistical analysis and to determine differentially expressed miRNAs, median normalized signal intensities were log2 transformed and subjected to Student's paired t-test using Microsoft Excel 2010. We found 23 miRNAs to be significantly differentially expressed in serum before versus after chemotherapy (P ≤ 0.01, Fig. 2B). The three most significantly differentially expressed miRNAs were miRNA-320c (P = 0.0002), miRNA-1275 (P = 0.0005), and miRNA-3663-3p (P = 0.0006), among which miR-320c and miR-1275 were downregulated and miR-3663-3p was upregulated after chemotherapy.

The hybridization protocol, raw and normalized data are provided in NCBI's Gene Expression Omnibus (GEO, Series accession number GSE57570).

## 3 Discussion

Our data show that miRNA expression profiles in serum change in the course of treatment of hematologic diseases. Overall miRNA levels in patient sera were elevated prior to chemotherapy and compared to healthy controls, in accordance with aberrant miRNA expression and disease-specific miRNAs which have been described in various hematologic disorders such as AML, MM [6] and ALL [7]. However, we observed that the number of detected miRNAs correlated with the patients' blood cell count. This is in line with previously described findings, that the majority of circulating miRNAs originates from blood cells and that disturbance in blood cell counts can influence miRNA expression in serum [8]. Taking this into account, we normalized the expression profiles in order to identify specific miRNAs that significantly changed before versus after therapy. Normalization of miRNA data from serum samples is not trivial as standard normalization methods are not applicable here. Quantile normalization which is commonly used for processing of gene expression data is critical because miRNA expression data sets contain comparatively few data points [9]. Normalization using small nuclear RNAs such as RNU6B is not recommended as their expression was found to be dysregulated under pathological conditions [10] or they are not detectable in serum [11]. Another common method is the normalization to endogenous reference miRNAs such as miRNA-16, but it has been shown that the expression of this reference miRNA can be unstable or influenced by hemolysis [8], [12]. Thus, we decided to normalize our data to the array median prior to identification of miRNA candidates. Subsequent statistical analysis revealed 23 significantly differentially expressed miRNAs, of which miRNA-320c showed the highest significance and was chosen for further analysis such as qRT-PCR, target prediction and biological function in HSPCs [5].

## Conflict of interest

The authors declare that Yvonne Diener, Ute Bissels and Andreas Bosio are employed at Miltenyi Biotec GmbH. Wolfgang Wagner is involved in the Cygenia GmbH. No further competing interests exist.

## References

[1] Walenda T., Bokermann G., Jost E., Galm O., Schellenberg A. (2011). Serum after autologous transplantation stimulates proliferation and expansion of human hematopoietic progenitor cells. PLoS One.

[2] Keller A., Leidinger P., Bauer A., Elsharawy A., Haas J. (2011). Toward the blood-borne miRNome of human diseases. Nat. Methods.

[3] Grasedieck S., Sorrentino A., Langer C., Buske C., Dohner H. (2013). Circulating microRNAs in hematological diseases: principles, challenges, and perspectives. Blood.

[4] Bissels U., Bosio A., Wagner W. (2012). MicroRNAs are shaping the hematopoietic landscape. Haematologica.

[5] Walenda T., Diener Y., Jost E., Morin-Kensicki E., Goecke T.W. (2015). MicroRNAs and metabolites in serum change after chemotherapy: impact on hematopoietic stem and progenitor cells. PLoS One.

[6] Undi R.B., Kandi R., Gutti R.K. (2013). MicroRNAs as haematopoiesis regulators. Adv. Hematol..

[7] Chavali S., Bruhn S., Tiemann K., Saetrom P., Barrenas F. (2013). MicroRNAs act complementarily to regulate disease-related mRNA modules in human diseases. RNA.

[8] Pritchard C.C., Kroh E., Wood B., Arroyo J.D., Dougherty K.J. (2012). Blood cell origin of circulating microRNAs: a cautionary note for cancer biomarker studies. Cancer Prev. Res. (Phila).

[9] Tiberio P., Callari M., Angeloni V., Daidone M.G., Appierto V. (2015). Challenges in using circulating miRNAs as cancer biomarkers. Biomed. Res. Int..

[10] Benz F., Roderburg C., Vargas Cardenas D., Vucur M., Gautheron J. (2013). U6 is unsuitable for normalization of serum miRNA levels in patients with sepsis or liver fibrosis. Exp. Mol. Med..

[11] Wang K., Yuan Y., Cho J.H., McClarty S., Baxter D. (2012). Comparing the MicroRNA spectrum between serum and plasma. PLoS One.

[12] Kirschner M.B., Kao S.C., Edelman J.J., Armstrong N.J., Vallely M.P. (2011). Haemolysis during sample preparation alters microRNA content of plasma. PLoS One.

